# Co-evolution of risk and cooperation in climate policies under wealth inequality

**DOI:** 10.1093/pnasnexus/pgae550

**Published:** 2024-12-09

**Authors:** Jorge M Pacheco, Francisco C Santos

**Affiliations:** INESC-ID, IST-Taguspark, 2744-016 Porto Salvo, Portugal; ATP-Group, P-2744-016 Porto Salvo, Portugal; INESC-ID, IST-Taguspark, 2744-016 Porto Salvo, Portugal; ATP-Group, P-2744-016 Porto Salvo, Portugal; Instituto Superior Técnico, Universidade de Lisboa, IST-Taguspark, 2744-016 Porto Salvo, Portugal

**Keywords:** collective action, global warming, governance of the commons, environmental agreements, evolutionary game theory

## Abstract

Worldwide cooperation is necessary to mitigate the effects of climate change. Many previous investigations employed the so-called collective risk dilemma, where the risk of losing everything whenever a target is not met was fixed from the outset, rendering predictions dependent on snapshot values assumed for this parameter, whose importance was found to be paramount. Here, we couple risk with the overall success of mitigation, investigating the co-evolution of risk and cooperation in a world where countries are partitioned in two different wealth classes, allowing us to further assess the impact of wealth inequality and homophily on the co-evolutionary dynamics. We show that the stochastic dynamics is dominated by a global attractor, typically located in a region of low risk, where most developed countries cooperate most of the time while developing countries cooperate to a lesser extent. This scenario assumes no homophily which, when moderate, can contribute to increase overall cooperation, more so when combined with the presence of a small fraction of developing countries that opt for an unconditional cooperative behavior.

**Significance Statement**

The willingness of countries to cooperate in mitigating the effects of climate change has been shown to depend significantly on the global perception of the risk of failure. In general, one expects that under widespread cooperation, the overall perception of risk will tend to decline, which may act to increase the temptation for free-riding, thus leading to a decline of cooperation. Here, we establish such an interdependence between risk and cooperation and investigate whether it is detrimental or beneficial to overcome climate change. We show how developed countries steer global cooperation to remain stable at low risk and how the detrimental effects of homophily may be overcome by ensuring that a few developing countries cooperate unconditionally.

## Introduction

The collective risk dilemma (CRD) has been widely employed (1–20) in investigating the feasibility of globally reducing greenhouse gas emissions (GHGE). It is a threshold public goods game (PGG) (21) where overall cooperation may ensure success in taming GHGE, whereas failure to cooperate by most parties will induce a tragedy of the commons. In the CRD, mitigation efforts imply a cost, whose benefits may become available to all. Thus, the temptation to *free-ride* on the benefits produced by others at their own expense is an inescapable component of the model. On the contrary, the existence of a threshold leads to scenarios where individuals may cooperate in vain, in which case free riders may also lose all their endowments. As a result, individual cooperation does not ensure success.

An important parameter of the model is global risk perception. Both behavioral experiments and theoretical work predict that risk plays a central role in escaping the tragedy of the commons embodied in the CRD. However, up to now risk has been used as an exogenous parameter (1–20) that remains fixed (for an exception, see (19), while overall behavior evolves: Conceptually, increasing risk reflects the overall awareness of the increasing likelihood of failing some predefined target (say, become carbon neutral by 2050).

Clearly, risk perception is not expected to remain fixed as countries’ policies and policy implementations evolve in time. Behavioral experiments provide some clues in this direction (in line with previous considerations), as one observes that when individuals have repeated chances to contribute to the public good while being aware of group performance at all times, good group performance often leads to decline in contributions in the last rounds (1–3) whereas bad group performance prompts individuals to increase their subsequent contributions (18) (see also (22, 23)). Consequently, one expects that low levels of cooperation will act to increase risk perception, whereas high levels of cooperation will act to decrease risk perception.

Besides the expected behavior at low and high cooperation levels, no data exist on how risk perception changes as a function of cooperation (12, 19). Moreover, the fact that in the CRD not every act of cooperation leads to the production of a public good suggests that risk perception may not depend straightforwardly on the amount of cooperation (or defection) in the population (in the Supplementary Information [SI], we discuss in more detail the threshold dependence of the risk–cooperation coupling).

**Fig. 1.**
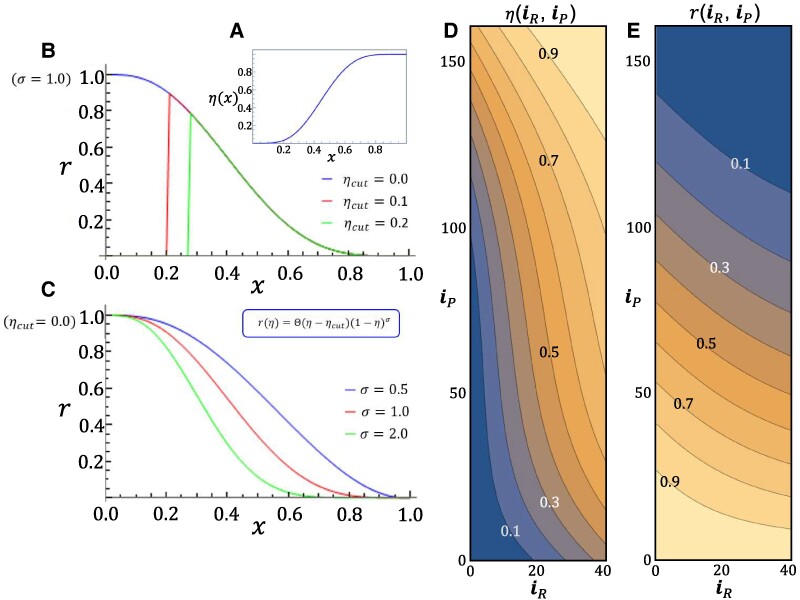
Risk dependence on collective success. A) The success of collective action in the CRD is best measured by the group success η(x), a function of the fraction x (or number, see below) of cooperators in the population, exhibiting the *S*-shape dependence illustrated (here, for convenience, we denote by x (0≤x≤1) the fraction of cooperators in the population, see below). Risk perception *r* is coupled to group success *η* via the two-parameter functional relation defined in Equation 1. The dependence of risk on the parameters $\left\{\eta_{\mathrm{cut}},\sigma \right\}$ is illustrated in B) (for fixed σ=1.0) and C) (for fixed $\eta_{\mathrm{cut}}=0.0$), respectively, as a function of x; D) Group success $\eta (i_{R},i_{P})$, defined in Equation 2, is drawn for the problem under investigation here, where two wealth classes—*rich* and *poor*—are considered (see main text and Materials and methods for details); the result is a contour plot on the configuration simplex defined by the variables $i_{R}\;(i_{P})$, respectively, the number of *rich* (*poor*) cooperators in the population of finite size *Z*. For each point in the simplex, associated with a possible configuration of the population, *η* will assume a specific value. E) For the present model, $\eta \to \eta (i_{R},i_{P})$; thus, $r\to r(i_{R},i_{P})$ leading to the contour illustrated for typical parameter values of the present model. Note, in particular, that about 40% of the upper area of the simplex configures a low-risk regime in which *r* < 0.2, a qualitative threshold above which group success was found to increase significantly in Ref. (10) (for the same parameters). Model parameters used: $Z=200,\;Z_{R}=40\;(20\text{\%}),\;Z_{P}=160\;(80\text{\%}),\;s=4,\;\;c=0.1,\;\left\langle b\right\rangle =1,\;\;N=6,\;\;M=3,\;\sigma =0.5,\;\eta_{\mathrm{cut}}=0.\;$ Note, finally, that the parameters $\left\{\eta_{\mathrm{cut}},\sigma \right\}$, similar to *η*, may be wealth class dependent (see Fig. 2 and Materials and methods).

Therefore, in the following we couple global risk perception with the overall success in avoiding a tragedy of the commons. A natural measure of this success in the CRD (10, 24, 25) is the quantity η∈[0,1], the so-called group success, a (nonlinear) function that returns the probability of overcoming the threshold in the CRD (defined below, Equation 2). It is a global, population-wide quantity which depends on the number of countries that cooperate at a given time, as well on the specificities of the PGG under study (see SI, Section 4 for explicit examples). The quantity *η* is a growing function of the total number of cooperators in a finite population of size *Z* and is illustrated in Fig. 1A, evidencing a characteristic *S*-shape and in Fig. 1D for the model we shall consider here, where we include wealth inequality in the population by distinguishing between *rich* ($i_{R}$, developed) and *poor* ($i_{P}$, developing) countries (see below). Denoting by $i_{R}$ ($i_{P}$), the number of *rich* (*poor*) cooperators in the population of finite size *Z*, $\eta (i_{R},i_{P})$ becomes a function of two variables. In this work, we establish a direct coupling between risk *r* and cooperation via $\eta (i_{R},i_{P})$ through the following nonlinear two-parameter function of $\eta \;(\{\eta_{\mathrm{cut}},\sigma \}\geq 0)$


(1) $$r(\eta )=\Theta (\eta -\eta_{\mathrm{cut}})(1-\eta {)}^{\sigma }$$

where Θ(ξ) denotes the Heaviside step function (Θ(ξ)=0 if ξ<0 and 1 otherwise). The intuition for this coupling form is illustrated in Fig. 1B, C, and E. When the population is dominated by defectors ($i_{R}+i_{P}∼0$ or x∼0 in Fig. 1), η∼0 and one expects r∼1. On the contrary, when cooperators dominate ($i_{R}+i_{P}∼Z$ or x∼1 in Fig. 1), η∼1 and one expects that r∼0. Between these two limiting cases, the rate of transition from 1 to 0 with increasing cooperation is controlled by the parameter σ: For σ=1, *r* will decrease linearly with *η*; σ>1 leads to a convex decrease of *r* with *η*, whereas σ<1 leads to a concave decrease. This said, it is noteworthy that the nonlinear dependence of *η* on the amount of cooperation will ultimately result in the general nonlinear dependence of *r* on cooperation shown in Fig. 1. The other parameter in Equation 1, $\eta_{\mathrm{cut}}$, is here introduced to account for the possibility of occurrence of a “point of no-return,” that is, a critical value of *η* below which risk becomes zero (cooperation is no longer worthwhile). Needless to say, this is one possible way to define an explicit coupling between risk and cooperation. In the following, we will show how global cooperation depends sensitively on *σ* and, surprisingly, much less so on $\eta_{\mathrm{cut}}$.

As a result, we shall investigate, in the framework of Evolutionary Game Theory of Finite Populations (see Materials and methods), the stochastic co-evolutionary dynamics (26) of cooperation under wealth inequality when risk becomes a (nonlinear) function of the amount of successful cooperation in the population: $r\to r[\eta (i_{R},i_{P})]$, as illustrated in Fig. 1. Indeed, the risk dependence on collective success embodied in Equation 1 leads to an entirely new formulation of the co-evolution of risk and cooperation and thus to a new framework in which to investigate the role of risk in the governance of risky commons.

Previous investigations carried out at fixed risk (4, 10) show that in the absence of risk the CRD becomes an N-person prisoner’s dilemma where cooperation collapses and the tragedy of the commons ensues. High (fixed) risk, instead, opens a window of opportunity for cooperation to thrive (4). Clearly, the nonlinear coupling between risk and cooperation illustrated in Fig. 1 allows for countries to experience all possible scenarios, ranging from the easy temptation to free-ride on the effort of others at low risk to the fear of missing the target at high risk—or, in extreme situations where cooperation eventually falls below a point of no-return (determined by $\eta_{\mathrm{cut}}$), to lose all hope of success.

Let us consider a well-mixed population of finite size *Z* where individuals can either cooperate (*C*) or defect (*D*) and where there is an asymmetric distribution of *rich* (mimicking a number $Z_{R}$ of developed countries) and *poor* (mimicking a number $Z_{P}=Z-Z_{R}$ of developing countries) players, and let us investigate the behavioral interplay between *rich* and *poor* in time, regarding their willingness to cooperate or not (4, 10). All values $\left\{Z,\;Z_{R}\;,\;Z_{P}\right\}$ remain fixed throughout evolution, though not their behavior (*C* or *D*). A population configuration i is uniquely identified by the pair of numbers of cooperators $i\equiv \{i_{R},i_{P}\}$ ($0\leq i_{R}\leq Z_{R}$ and $0\leq i_{P}\leq Z_{P}$) in that configuration. Players are randomly sampled from the population and organize into groups of size *N*. A group configuration j can be similarly identified by the corresponding pair of numbers of cooperators $j\equiv \{\;j_{R},\;j_{P}\}$ ($0\leq j_{R}\leq N$ and $0\leq j_{P}\leq N-j_{R}$) in that group. Each *rich* (*poor*) individual starts with an initial endowment $b_{R}$ ($b_{P}$) with $b_{R}=4\;b_{P}$ (10). Part of these endowments may be used (or not) by individuals to contribute to reduce GHGE in their own group. *Rich* (*poor*) *C*s contribute some part $c_{R}$ ($c_{P}$) of their endowment to help solve the group task, while *Defectors* (*D*s, *rich* and *poor*) do not contribute anything to solve the group task. Hence, the endowment is directly related to what each participant will lose if the next target is not met: *C*s will lose $b_{R|P}(1-c_{R|P})$, whereas *D*s will lose the entire endowment $b_{R|P}$. If the total amount of contributions in a group satisfies Δ≥0 (with $\Delta =j_{R}c_{R}+j_{P}c_{P}-M\left\langle c\right\rangle \;$ and $Z\left\langle c\right\rangle =Z_{R}c_{R}+Z_{P}c_{P}$), that is, if it meets or exceeds a predefined threshold M⟨c⟩―where 0<M≤N is the group threshold and ⟨c⟩ is the average cost of cooperation, then the target will be met. Otherwise, with a probability r(η), dependent on the average group success *η*, every individual in the group will lose whatever they have. We use multivariate hypergeometric sampling to compute the group success *η*, which corresponds to the (average) fraction of groups that reach a total of M⟨c⟩ in contributions for a given population configuration, thereby producing a public good


(2) $$\eta (i_{R},i_{P})={\left(\begin{array}{c} Z\\ N \end{array}\right)}^{-1}\sum_{\;j_{R}=0}^{N}\sum_{\;j_{P}=0}^{N-j_{R}}\left(\begin{array}{c} i_{R}\\ \;j_{R} \end{array}\right)\left(\begin{array}{c} i_{P}\\ \;j_{P} \end{array}\right)\left(\begin{array}{c} Z-i_{R}-i_{P}\\ N-j_{R}-j_{P} \end{array}\right)\Theta (\Delta )$$

where *Δ* and Θ were already defined before.

The number of individuals adopting a given strategy will evolve in time according to a stochastic birth–death process combined with the pairwise comparison rule (27), which describes the social dynamics of *rich C*s, *poor C*s, *rich D*s, and *poor D*s in a ﬁnite population. Under pairwise comparison, each individual of strategy *X* adopts the strategy Yof another member of the population, with probability given by the Fermi function $[1+e^{\beta (\;f^{X}-f^{Y})}{]}^{-1}$, where *β* controls the intensity of selection. The fitness $f^{X}$ of an individual adopting a strategy *X* corresponds to the average payoff of that strategy in the population.

Individuals may further exhibit a variable degree of homophily, which acts to limit those that constitute each one’s pool of influence. In the absence of homophily, individual *Y* is chosen with uniform probability between the other Z−1 individuals. With increasing homophily *h*, the random choice of *Y* will become increasingly biased toward choosing individuals from the same wealth class as *X*. We consider a homophily parameter 0≤h≤1 that will allow us to explore the gradual transition from no homophily (h=0) to full homophily (h=1, see Materials and methods and SI for specific details of the update rule, in particular whenever homophily is explicitly taken into consideration). It is noteworthy that, independently of *h*, pairwise comparison between *X* and *Y* always involves a fitness comparison.

Each player in a group knows what all other members of the group will do, such that decisions and achievements of others influence one’s own decisions (10). In particular, decisions taken by the *poor* can be potentially influenced by the actions and achievements of the *rich* (and vice versa) depending on the homophily parameter *h*. Such an influence dynamics occurs in the presence of action errors (24) as well as other stochastic effects, such as random exploration of the strategy space (also known as behavioral mutations) (28).

**Fig. 2.**
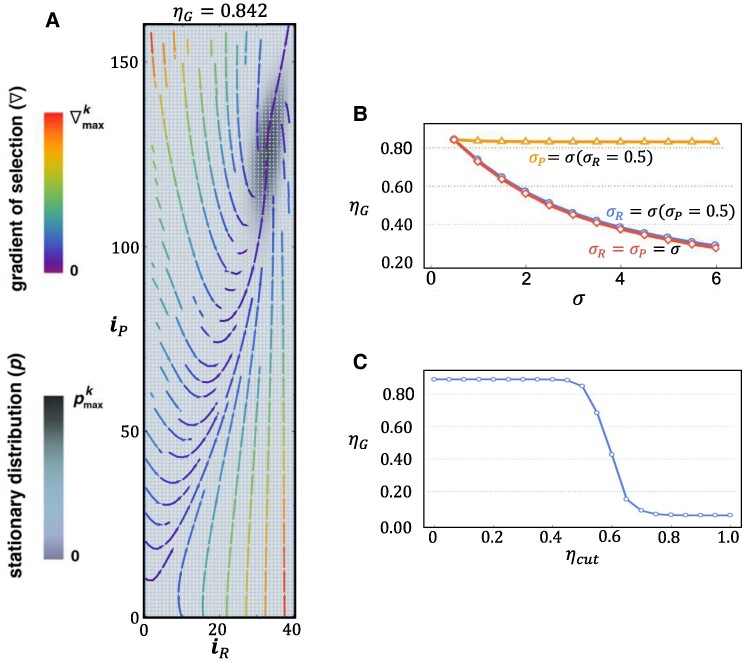
Co-evolution of cooperation and risk in the CRD. A) Stationary distribution and gradient of selection in the absence of homophily (h=0). The panel contains all possible configurations of the population (a total of $\left(1+Z_{R})(1+Z_{P}\right)$), each specified by the number of *rich* ($i_{R}$) and *poor* ($i_{P}$) and represented by a gray-colored dot. Darker dots represent those configurations in which the population spends more time, thus providing a contour representation of the stationary distribution. The arrows show the so-called gradient of selection (∇), which provides the most likely direction of evolution from a given configuration. We use a color code in which red lines are associated with higher rates of change. The co-evolutionary dynamics is dominated by an attractor—centered at a configuration of the population in which ∼83% of *rich* and ∼78% of *poor* contribute to mitigate — and into which the population configuration converges in a stochastic sense, determining a final population average group success $\eta_{G}\;$ (see Materials and methods) of ∼84%. B) $\eta_{G}$ is plotted as a function of the parameter *σ* controlling the rate of change of *risk* as a function of group success *η* (for fixed $\eta_{\mathrm{cut}}=0.0$). Here, we allow *σ* to assume different values for *rich* ($\sigma_{R}$) and *poor* ($\sigma_{P}$) and compute $\eta_{G}\;$ for situations in which we vary only $\sigma_{R}\;$ (blue), only $\sigma_{P}$ (orange), and both $\sigma_{R}$ and $\sigma_{P}\;$ (red). Clearly, the overall co-evolutionary dynamics is controlled by the value of $\sigma_{R}$. C) Role of $\eta_{\mathrm{cut}}$ on the co-evolutionary dynamics: We keep $\sigma_{R}=\sigma_{P}=0.5$ and compute $\eta_{G}$ for different values of $\eta_{\mathrm{cut}}$. Results show that overall cooperation will collapse only for values of $\eta_{\mathrm{cut}}$ for which there is no risk in most of population configurations. Model parameters: $Z=200,\;Z_{R}=40,\;Z_{P}=160,\;\;c_{R}=4c_{P}=0.25,\;b_{R}=4b_{P}=2.5,\;N=6,\;\;M=3,\;\beta =3,\;\mu =Z^{-1},\;h=0,$  $\eta_{\mathrm{cut}}=0,\;\;\sigma_{R}=\;\sigma_{P}=0.5.\;$ Plot limits: $\;p_{\max}^{k}=5.3\times 10^{-3};\;\nabla_{\max}^{k}=16.14\times 10^{-2}$.

Analysis of the fully coupled dynamics on the entire configuration requires the plot of the two-dimensional simplex (see Figs. 1D, E and 2A), in which the *y*-axis (*x*-axis) portrays the number of *C*s among the *poor* (*rich*) sub-populations. In breaking the population into two wealth classes, the *poor* comprise 80% of the population, while the *rich* constitute the remaining 20% (10). For a population size of Z=200, the phase space simplex will encompass a (41×161) cell rectangle, as shown.

We shall finally consider several possible threshold PGG scenarios in what concerns the cost of mitigation and contributions to mitigate: We start by generally assuming that the cost of mitigation of *rich* countries is *s* times higher than the cost of mitigation of *poor* countries ($c_{R}=s\;c_{P}$). In Ref. (10), we made s=4, in which case the cost of mitigation is proportional to the wealth of the country, given that $b_{R}=4\;b_{P}\;$ (further details in SI). World Bank data ((18) and references therein) lead to values of s≈3, implying that the parametrization of Ref. (10) configures a scenario in which the cost of cooperation for the *rich* is high. We shall investigate here the case s=4 which enables a direct comparison to existing results at fixed risk (10) leaving to the SI the detailed analysis of the evolutionary dynamics for other values of *s*.

## Results

In Fig. 2A, we plot the co-evolutionary dynamics on the entire simplex. Two quantities are plotted in panel A: the stationary distribution (*p*) of the co-evolutionary dynamics (see Materials and methods for details), represented by (gray color coded) shaded circles, and the gradient of selection (∇), represented by (color coded) flow arrows providing, at any point in phase space, the most likely direction of evolution from that point. We make $\eta_{\mathrm{cut}}=0.0$ (see Fig. 1) to allow for full exploration of *risk* and fix the exponent *σ* to $\sigma_{R}=\sigma_{P}=0.5$ (see Discussion in connection to Fig. 2B below).

We find that the global co-evolutionary dynamics is dominated by an attractor at high values of both $i_{R}$ and $i_{P}$, leading to a population average group success of $\eta_{G}=84\text{\%}$ (see Materials and methods). On average, the majority of *rich* (∼83%) and of *poor* (∼78%) contribute to mitigate.

It is noteworthy that the co-evolutionary dynamics leads to nontrivial trajectories of approaching the center of the attractor (the dark shaded area of the simplex, centered at $i_{R}^{*}\approx 33,\;i_{P}^{*}\approx 127$). Indeed, starting from configurations (compared with $\left(i_{R}^{*},i_{P}^{*}\right)$) with excess of *rich* and deficit of *poor* (excess of *poor* and deficit of *rich*), the co-evolutionary dynamics leads to an increase (decrease) of *poor* (*rich*) that overshoots (undershoots) the attractor location before converging (in a stochastic sense) slowly—as indicated by the arrow colors—toward its center. Furthermore, it is noteworthy that in the vicinity of $i_{R}=i_{P}=0$, there is a hint of what looks as a repeller (in fact a stochastic analog of a saddle point), which becomes more clearly defined for other model parameters (see SI for concrete examples).

Figure 2B shows the role played by exponent *σ* on the resulting co-evolutionary dynamics assessed in terms of $\eta_{G}$. Clearly, the results rely almost entirely on the value $\sigma_{R}\;$ associated with the *rich* wealth class. Indeed, by allowing for a different risk dependence on *η* — spanning both the concave and convex dependence of *r* on *η* — for *rich*  $(\sigma_{R})\;$ and for *poor*  $\left(\sigma_{P}\right)$, the results of Fig. 2B clearly show that fixing $\sigma_{R}$ while varying $\sigma_{P}$ (orange curve) leads to no qualitative change of $\eta_{G}$. Varying $\sigma_{R}$ while keeping $\sigma_{P}$ constant (blue curve) as well as varying both $\sigma_{R}$ and $\sigma_{P}$ (red curve) leads to sizable (and similar) changes in $\eta_{G}$. Intuitively (see Fig. 1), increasing $\sigma_{R}$ implies imposing a steeper decline of risk with increasing *η* which acts to reduce $\eta_{G}$.

The scenario portrayed in Fig. 2A remains qualitatively unchanged as we also change the parameter $\eta_{\mathrm{cut}}$. This is shown in Fig. 2C, where $\eta_{G}$ remains stable for changes of $\eta_{\mathrm{cut}}$ up to $\eta_{\mathrm{cut}}=0.6.\;$ Naturally, this threshold dependence (here obtained for $\sigma_{R}=\sigma_{P}=0.5$) depends on the value of *σ*. Finally, the behavior illustrated in Fig. 2A remains qualitatively similar if we change population size as well as if we change the mitigation cost ratio *s* introduced before (see SI for details).

The co-evolutionary dynamics emerging from Fig. 2 seem counterintuitive, given the risk distribution shown in Fig. 1E (obtained for the same model parameters) which shows that the attractor of the co-evolutionary dynamics in Fig. 2A is centered at configurations where risk perception lies below 10%. More so if we take into consideration that most of the stationary distribution is located around the attractor—which means that the population spends most of the time in regions of low risk. Clearly, the results of Fig. 2 cannot be anticipated based on the results obtained in Ref. (10) where risk was fixed from the outset and remained constant throughout the (strategy) evolutionary process. Indeed, when compared with results for the same parameters in Ref. (10), we would need (fixed) risk values over 25% to achieve comparable values for $\eta_{G}$. The present result, in fact, stems from the (nonlinear) dynamic coupling between risk and group success.

**Fig. 3.**
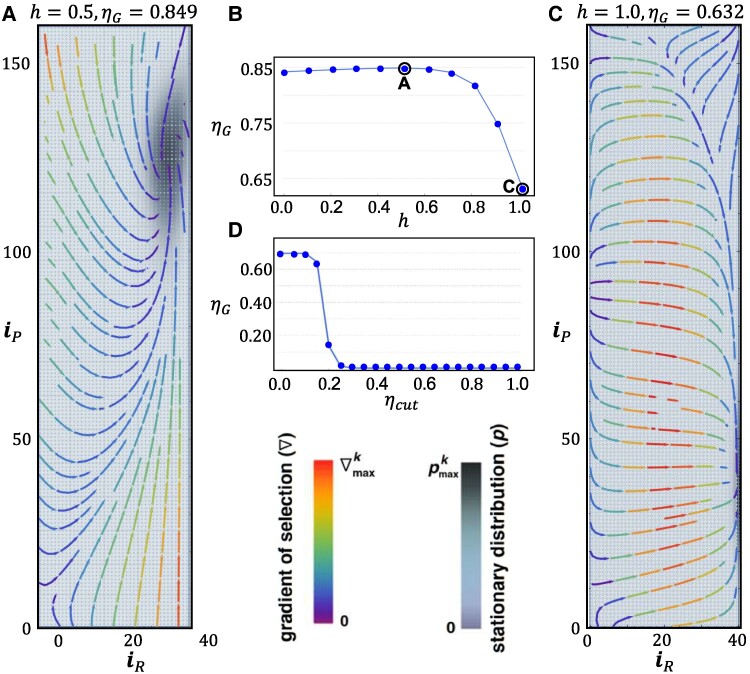
Co-evolution of cooperation and risk under growing homophily. A) Phase portraits of the stationary distribution and gradient of selection for h=0.5. The behavior is very similar to that shown in Fig. 2A for h=0 and leads to a <1% higher value for $\eta_{G}$. B) $\eta_{G}$ evidences a nonmonotonic behavior as a function of *h*: It grows marginally up to h=0.5, declining by 25% as *h* increases further to h=1. C) Phase portraits of the stationary distribution and gradient of selection for h=1.0. When all population exhibits a full homophilic behavior, the *rich* still cooperate, while only about 25% of the *poor* remain cooperative, on average. D) The dependence of $\eta_{G}$ on the parameter $\eta_{\mathrm{cut}}$ (see Fig. 1) shows a more sensitive dependence for h=1 when compared with h=0 (see Fig. 2C), as in this case group success declines to zero already when $\eta_{\mathrm{cut}}=0.25.$ Model parameters: same as in Fig. 2. Plot limits: $p_{\max}^{k}=3.7\times 10^{-3}\;(A)\;\mathrm{and}\;\;21.9\times 10^{-3}\;(C);\;\nabla_{\max}^{k}=9.27\times 10^{-2}(A)\;\mathrm{and}\;4.49\times 10^{-2}\;(C)$.

This same coupling also explains the resilience of overall cooperation (compared with the fixed risk scenario of Ref. (10)) when homophily increases in the population. In Fig. 3, we illustrate the behavior of the population as a function of homophily: We keep the same model parameters of Fig. 2 and change the homophily parameter *h* from 0 to 1 (Fig. 3B). We obtain a nonmonotonic behavior of $\eta_{G}$, which shows a marginal increase of <1% from h=0.0 to a maximum at h=0.5 (phase portrait plotted in Fig. 3A), followed by a decrease of 25% up to h=1 (phase portrait plotted in Fig. 3C). The phase portraits shown in Fig. 3A and C show that as the behavior becomes 100% homophilic, the *rich* still cooperate, while one witnesses a sharp decline of cooperation by the *poor*, where only about 25% remain cooperative, on average.

This behavior contrasts sharply with what was obtained under fixed risk in Ref. (10), where cooperation was shown to collapse when homophily was present, even at high risk. When combined, the results shown in Figs. 2 and 3 suggest that, under variable risk, the *rich* countries are steering cooperation in the CRD. This feature also provides an intuition for why $\eta_{G}$ increases (albeit marginally) with increasing *h*: Indeed, for moderate values of *h*, *rich* are more effective in influencing each other into cooperation which, in face of their leading role in the overall dynamics, fosters higher levels of global cooperation.

High homophily is typically associated with high levels of political polarization. It is, however, hard to imagine scenarios in which h=1 is realized in practice. In this context, the message from Fig. 3B is encouraging, as even for polarization levels associated with h≈0.7 the overall level of cooperation is not compromised. Finally, Fig. 3D shows that, under homophily, the co-evolutionary dynamics becomes more sensitive to the parameter $\eta_{\mathrm{cut}}$ (compare, e.g. with Fig. 2C).

**Fig. 4.**
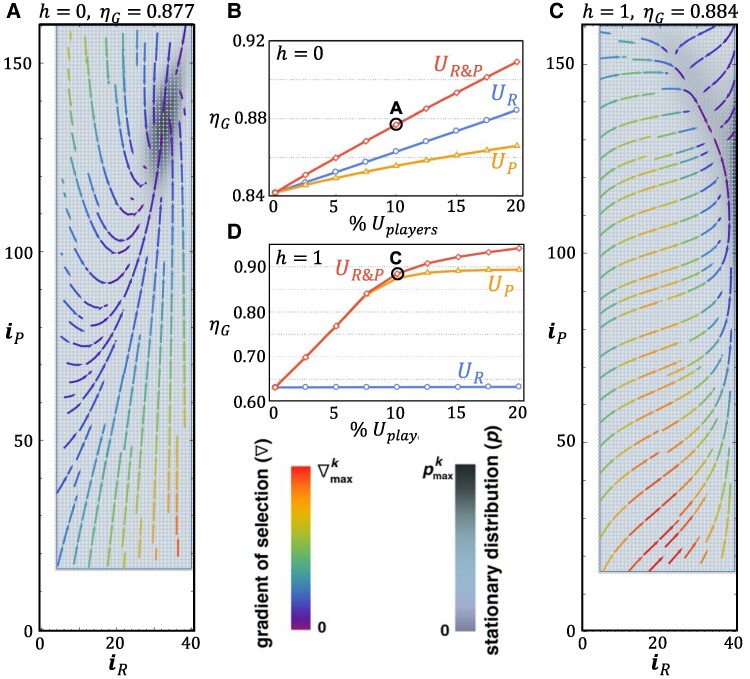
Co-evolutionary dynamics in the presence of unconditional behavior. A) Phase portraits of the stationary distribution and gradient of selection for h=0 when 10% of *poor* and 10% of *rich* exhibit unconditional cooperative behavior ($U_{P|R}$). The peak of the stationary distribution occurs for the configuration $i_{R}^{*}\approx 33,\;i_{P}^{*}\approx 135\;$ which, when compared with the scenario of Fig. 2A, shows that only the number of *poor* cooperators actually increases (by 8). B) $\eta_{G}$ is plotted as a function of the fraction of $U_{P|R}\;$ in the population for h=0. We use the same color convention as in Fig. 2B. In the absence of homophily, $U_{R}$ acts to effectively increase the number of *poor* that cooperate. C) Same as A for h=1. When $U_{P}=10\text{\%}$, the number of *poor* that cooperate significantly increases and, as a result, so does $\eta_{G}$. D) Same as B for h=1, where we maintain the color conventions. Comparison with B shows that, under full homophily, below 10% only $U_{P}$ contribute to increase $\eta_{G}$, whereas for higher fractions of unconditional players, both *rich* and *poor* contribute to increase $\eta_{G}$; it is noteworthy that, despite full homophily, the increase of cooperation of one wealth class still determines a higher rate of successful production of public goods, which acts to increase $\eta_{G}$. Model parameters: same as in Fig. 3. Plot limits: $p_{\max}^{k}=6.5\times 10^{-3}\;(A)\;\mathrm{and}\;\;6.2\times 10^{-3}\;(C);\;\nabla_{\max}^{k}=19.74\times 10^{-2}(A)\;\;\mathrm{and}\;\;6.65\times 10^{-2}\;(C)$.

Another important feature of present-day behavior in connection to climate change is related to the urgency that some countries impose on their agenda toward mitigation of GHGE. Such behavior may translate, in our model, into allowing that some members of the population assume an unconditional behavior, in the sense that such countries may not be influenced by any of their peers. In the following, we study the impact of such unconditional behavior under co-evolutionary risk and cooperation. To this end, we fix, from the outset, a predefined number of individuals, of each class, that behave as unconditional cooperators, that is, always contribute to mitigate GHGE. In doing so, we effectively reduce the phase space (simplex) in which evolutionary dynamics takes place. We denote these unconditional cooperators as $U_{P|R}$. The results are summarized in Fig. 4.

Clearly, the appearance of unconditional behavior leads to an increase of overall cooperation, irrespective of the degree of homophily. However, whereas for h=0 we obtain an increase of ≈4% in $\eta_{G}$ when we assign 10% of both $U_{P}$ and $U_{R}$ behaviors of the population (Fig. 4A), for h=1 the corresponding increase is ≈ 40% (Fig. 4C). The behavior of $\eta_{G}$ as a function of the fraction of $U_{P|R}$ individuals in the population is detailed in Fig. 4B for h=0 and in Fig. 4D for h=1. Not only the behavior is very different at the limiting values of *h*, but also the role played by $U_{P|R}$ individuals is very different in those limits. Indeed, for h=0 the contributions of $U_{P}$ and $U_{R}$ “add up” linearly to the total value of $\eta_{G}$; for h=1 only when more than 10% of the population (*rich* and *poor*) exhibit unconditional behavior do the $U_{R}$ play a sizable role (Fig. 4D): The higher the fraction of $U_{R}$, the more successful groups are in producing a public good, as such contributing to increase $\;\eta_{G}$. This synergistic effect results from the nonlinear coupling between *rich* and *poor*, which remains despite the fact that we are in the limit h=1.

In face of the results obtained so far (see, e.g. Figs. 2A and 3C), together with the phase portraits of Fig. 4A and C, the intuition is clear:

The major impact of unconditional behavior happens among the *poor*, as the majority of *rich* already cooperate under variable risk, and hence, the improvement due to the presence of $U_{R}$ is minor. Indeed, even when h=1 and the majority of the *poor* do not cooperate (Fig. 3C) all of the *rich* cooperate most of the time. Hence, and in particular when h=1, the impact of a few $U_{P}$ is sizeable, such that 10% of $U_{P}$ lead to an increase of *poor* that cooperate from ≈ 40 up to ≈ 135. In all cases, most *rich* cooperate.

## Discussion

In summary, by establishing a natural coupling between the overall level of successful cooperation (as measured by *η*) and risk perception regarding failure to meet predefined targets, we obtained a co-evolutionary dynamics that is dominated by an interior attractor, where the majority of developed countries are expected to cooperate in carbon mitigation, whereas developing countries will mitigate but to a somewhat lesser extent. The feedback between risk perception and overall cooperation places the global attractor, perhaps counterintuitively, in a region of low risk (and high success), where the population spends most of the time. Overall, most *rich* individuals cooperate most of the time, whereas *poor* individuals are more sensitive to the degree of homophily in the population. While in the absence of homophily the *rich* steer the dynamics, under full homophily cooperation among the *poor* benefits greatly from the decision, by a small fraction of *poor* individuals, to unconditionally cooperate. Under such circumstances, the population average group success can be even higher than in the absence of homophily, as shown in Fig. 4. In fact, the presence in the population of a small number of countries that cooperate unconditionally, mixed with a mild degree of homophily, may help maximizing the chances of success in GHGE mitigation. This configuration resembles, to some extent, the actual behavior of countries regarding Climate Change mitigation.

The coupling between risk and cooperation employed here shows that only populations that ignore risk most of the time will witness a collapse of cooperation, given the insensitivity of the results to $\eta_{\mathrm{cut}}$ (Fig. 2). Furthermore, the co-evolutionary dynamics is sensitive to the rate of decline of risk with increasing cooperation (*σ*) mostly for the developed countries, being largely insensitive to that rate for developing countries. At present, we believe that the conclusions stemming from many climate summits as well as the repeated appeals from many governments and also institutions — such as the United Nations — regarding the detrimental effects of Climate Change have led to a concrete awareness of countries (and citizens) regarding the need to reduce GHGE. Such an awareness would favor, in our model, a concave (σ<1) rather than a convex (σ>1) dependence of risk on group success. These are promising news, as our model shows that σ<1 favors overall cooperation. Furthermore, the present model also suggests that cooperation can emerge for population configurations where (adaptive) risk is, on average, moderate.

Investigations of Climate Change mitigation carried out to date involving the CRD or similar PGG dilemmas (1–20) have all considered values for the group size satisfying N≤10. This is related to the well-known fact that cooperation in PGG dilemmas declines rapidly with increasing group size (21). From this perspective, the global climate summits that have been taking place during the last decades, which translate into groups of size N∼Z∼200, configure a daunting task with no positive solution to be expected from a game theoretical perspective. This grim prospect remains, even when institutions designed to enforce agreements are setup at a global scale (24). On the contrary, approaches such as this one that advocate a bottom-up approach, wherein cooperation is sought in small groups, eventually self-organizing into a population-wide cooperative dominance, lead to more positive prospects in what climate change mitigation is concerned. As shown already in (4), the well-mixed approach developed here provides a pessimistic scenario when compared with an explicit implementation where overlapping groups are formed as the result of an underlying network structure, which has been shown to act to increase the feasibility of cooperation. We expect that the present model will also provide a more positive perspective if simulated on a network, requiring a purely numerical approach. Work along these lines is in progress.

## Materials and methods

We consider a fixed population of Z individuals, $Z_{R}$ of which are considered *rich* (initial endowment $b_{R}$) and $Z_{P}\;$considered *poor* (initial endowment $\;b_{P}$) who, together, set up groups of size *N*, in which they engage in the CRD (10). Each individual adopts one of two behaviors: *C* or *D*. Following the discussion in the main text, we make $c_{R|P}=c\;b_{R|P}$ (we used the value c=0.1 for the cost factor *c*—see SI for other possibilities). Thus, the payoff of an individual playing in a group with $\left\{\;j_{R},\;j_{P}\right\}$  *C*s and $\;N-j_{R}-j_{P}$  *D*s can be written as $\;\Pi_{R|P}^{D}=b_{R|P}\{\Theta (\Delta )+(1-r[\eta (i_{R},i_{P})])[1-\Theta (\Delta )]\}$ and $\;\Pi_{R|P}^{C}=\Pi_{R|P}^{D}-c_{R|P}\;$ with $\Delta =c_{R}\;j_{R}+c_{P}\;j_{P}-M\left\langle c\right\rangle$, for *rich/poor D*s and *C*s, respectively. In the equations above, 0<M≤N is a positive integer, ⟨c⟩ is the average cost of cooperation, and r(η)∈[0,1] is defined in Equation 1, whereas the group success $\eta (i_{R},i_{P})$ is defined in Equation 2. The parameters $\;\left\{c_{R},\;c_{P},\;b_{R},\;b_{P},\;\left\langle b\right\rangle ,\;c<1\right\}$ are all positive real numbers, and we fix ⟨b⟩=1.0 ($Z\left\langle b\right\rangle =Z_{R}b_{R}+Z_{P}b_{P}$) (10). Finally, the fitness $f^{X}$ of an individual adopting a given strategy, *X*, is associated with the average payoff of that strategy in the population; similar to $\eta (i_{R},i_{P})$, the average payoff in the population configuration $\left\{i_{R},i_{P}\right\}$ can be computed using a multivariate hypergeometric sampling (without replacement) (see SI for explicit expressions). Evolution proceeds through a discrete sequence of stochastic birth–death steps combined with the pairwise comparison rule (27) (see main text for details) where an individual of strategy *X* adopts the strategy Y of another member of the population, with probability given by the Fermi function $[1+e^{\beta (\;f^{X}-f^{Y})}{]}^{-1}$. In the absence of homophily, the strategy *Y* is chosen at random with uniform probability. For a nonzero value of the homophily parameter *h*, individuals of the same wealth class are chosen with probability 1, whereas individuals of the other wealth class are chosen with probability 1−h; thus, when homophily is maximum, the choice occurs only among the individuals of the same wealth class (*rich* or *poor*) (see SI for explicit expressions). Additionally, we consider that, with a mutation probability *μ* (equal to 1/Z in all Figures except where explicitly indicated otherwise), individuals adopt a randomly chosen strategy (*C* or *D*). As the evolution of the population depends only on its actual configuration, the co-evolutionary dynamics can be described as a Markov process over a two-dimensional space. Its probability distribution function, $p_{i}(t)$, which provides information on the prevalence of each configuration at time *t*, obeys a *Master Equation* (see SI for details), a gain–loss equation involving the transition rates between all accessible configurations (29). The stationary distribution ${\bar{\;p}}_{i}$ is then obtained by reducing the *Master Equation* to an eigenvector search problem (29) (see SI for details). Another central quantity which portrays the overall evolutionary dynamics in the space of all possible configurations is the *gradient of selection*  $\;\nabla_{i}$. For each configuration $i\equiv \{\;i_{R},\;i_{P}\}$, we compute the most likely path the population will follow, resorting to the probability to increase (decrease) the number of individuals adopting a strategy $S_{k}$, $T_{i}^{S_{k}+}$ ($T_{i}^{S_{k}-}$) in each time-step. Additionally, for each possible configuration i, we compute η(i), the (average) fraction of groups that reach a total of M⟨c⟩ in contributions, that is, that successfully achieve the public good. Population average group success —$\;\eta_{G}\;$— is then computed averaging over all possible configurations i, each weighted using the corresponding stationary distribution: $\eta_{G}\;=\sum_{i}{\bar{\;p}}_{i}\eta (i)$.

## Supplementary Material

pgae550_Supplementary_Data

## Data Availability

There are no data underlying this work.
