# Supplementary material for: Co-evolution of risk and cooperation in climate policies under wealth inequality
Source: PNAS Nexus. 2024 Dec 9;3(12):pgae550. doi: 10.1093/pnasnexus/pgae550 (PMC11646703; doi:10.1093/pnasnexus/pgae550)
Supplement: pgae550_Supplementary_Data [file pgae550_supplementary_data.pdf]

Co-evolution of Risk and Cooperation in Climate Policies under Wealth Inequality

Jorge M. Pacheco[a,c,*], Francisco C. Santos[a,b,c]

[a] INESC-ID, IST-Taguspark, 2744-016 Porto Salvo, Portugal

[b] Instituto Superior Técnico, Universidade de Lisboa, IST-Taguspark, 2744-016 Porto Salvo, Portugal

[c] ATP-Group, P-2744-016 Porto Salvo, Portugal

* Corresponding author.

Email: jorgem.pacheco@gmail.com

This PDF file includes:

Supplementary text
Figures S1 to S6
SI References

Supplementary Text

In section 1 we provide additional details of the stochastic model employed, whereas sections 2 to 4 are devoted to provide an extensive analysis of the model behavior, exploring the dependence on its parameters, as well as providing additional information regarding some of the features discussed in the main text.

1. Model details

Let us consider a population of Z individuals. As stated in the main text, each individual adopts one of the two possible strategies $X\in\{C,D\}$ and belongs to one of two possible wealth classes $k\in\{R,P\}$. Let us assume there are Z_R *rich* (initial endowment b_R) and Z_P *poor* individuals (initial endowment $b_P < b_R$). These numbers will remain fixed. Individuals play the **CRD** in groups of size N. Following the discussion in the main text, and given that *rich* ***C***s contribute c_R whereas *poor* ***C***s contribute c_P, the payoff of an individual playing in a group in which there are j_R *rich* ***C***s, j_P *poor* ***C***s and $N - j_R - j_P$ ***D***s, can be written as

$$\Pi_{R|P}^{D} = b_{R|P}\{\Theta(\Delta) + (1 - r(x))\,[1 - \Theta(\Delta)]\}$$

and

$$\Pi_{R|P}^{C} = \Pi_{R|P}^{D} - c_{R|P}$$

with $\Delta = c_R\, j_R + c_P\, j_P - M\langle c\rangle$, for *rich/poor* ***D***s and ***C***s, respectively.

In the equations above, $\Theta(\mathrm{k})$ is the Heaviside function (that is, $\Theta(\mathrm{k}) = 1$ whenever $k \geq 0$, being zero otherwise), $0 < M \leq N$ a positive integer, $\langle b\rangle = 1$ is the average endowment ($Z\langle b\rangle = Z_R b_R + Z_P b_P$) where we made $b_R = 4\, b_P$ to reflect the fact that, 20% of wealthiest countries generate the same **GDP** as the remaining 80% of the countries worldwide. $r(\eta)\in[0,1]$ (the risk perception) is a two-parameter ($\{\eta_{cut},\sigma\}$) non-linear function of the group success defined in Equation **1** of main text, and $\eta(i_R,i_P)$, a real function of the number of cooperators (*rich* i_R and *poor* i_P) present in the population, defined in Equation **2** of main text. The parameters $c_R < b_R,$, $c_P < b_P$, $c < 1$ and $\langle b\rangle$ are all positive real numbers.

Finally, the fitness f_k^X of an individual adopting a given strategy X in a population of wealth class k, will be associated with the average payoff of that strategy in the entire population. This can be computed for a given configuration of strategies and wealth classes specified by $\boldsymbol{i} = \{i_R,i_P\}$, using a multivariate hypergeometric sampling (without replacement):

$$f_R^C(\mathbf{i}) = \binom{Z-1}{N-1}^{-1} \sum_{j_R=0}^{N-1} \sum_{j_P=0}^{N-1-j_R} \binom{i_R - 1}{j_R}\binom{i_P}{j_P}\binom{Z - i_R - i_P}{N-1-j_R-j_P}\Pi_R^C(j_R+1,j_P) \quad \text{(S1a)}$$

$$f_P^C(\mathbf{i}) = \binom{Z-1}{N-1}^{-1} \sum_{j_R=0}^{N-1} \sum_{j_P=0}^{N-1-j_R} \binom{i_R}{j_R}\binom{i_P - 1}{j_P}\binom{Z - i_R - i_P}{N-1-j_R-j_P}\Pi_P^C(j_R,j_P+1) \quad \text{(S1b)}$$

$$f_R^D(\mathbf{i}) = \binom{Z-1}{N-1}^{-1} \sum_{j_R=0}^{N-1} \sum_{j_P=0}^{N-1-j_R} \binom{i_R}{j_R}\binom{i_P}{j_P}\binom{Z-1-i_R-i_P}{N-1-j_R-j_P}\Pi_R^D(j_R,j_P) \quad \text{(S1c)}$$

$$f_P^D(\mathbf{i}) = \binom{Z-1}{N-1}^{-1} \sum_{j_R=0}^{N-1} \sum_{j_P=0}^{N-1-j_R} \binom{i_R}{j_R}\binom{i_P}{j_P}\binom{Z-1-i_R-i_P}{N-1-j_R-j_P}\Pi_P^D(j_R,j_P) \quad \text{(S1c)}$$

The number of individuals adopting a given strategy will evolve in time according to a stochastic birth–death process combined with the pairwise comparison rule (1, 2), which describes the stochastic social dynamics of *rich* ***C***s, *poor* ***C***s, *rich* ***D***s and *poor* ***D***s in a finite population. Under pairwise comparison, each individual of strategy X adopts the strategy Y of a randomly selected member of the population, with probability given by the Fermi function $\left(1+e^{\beta\left(f_k^X-f_l^Y\right)}\right)^{-1}$, for any wealth class $\{k,l\}\in\{R,P\}$, where β controls the intensity of selection. Additionally we consider that, with a mutation probability μ, individuals adopt a randomly chosen strategy. As the evolution of the system population only on its actual configuration, evolutionary dynamics can be described as a Markov process over a 2-dimensional space. Its probability distribution function, $p_{\mathbf{i}}(t)$, which provides information on the prevalence of each configuration at time *t*, obeys a *Master Equation* of the form

$$p_{\mathbf{i}}(t+\tau)-p_{\mathbf{i}}(t)=\sum_{\mathbf{i}'}\{T_{\mathbf{i}\mathbf{i}'}p_{\mathbf{i}'}(t)-T_{\mathbf{i}'\mathbf{i}}p_{\mathbf{i}}(t)\} \tag{S2}$$

a gain-loss equation that allows one to compute the evolution of $p_{\boldsymbol{i}}(t)$ given the transition probabilities per unit time τ from the configuration $\mathbf{i}$ to $\mathbf{i}'$ (2-4). The stationary distribution $\bar{p}_{\mathbf{i}}$ analysed in the main text, is obtained by making the left hand side equal to zero, which transforms Equation **S2** into an eigenvector search problem (3), namely, the eigenvector associated with the eigenvalue 1 of the transition matrix W (3) whose matrix elements W_{qp} are built from the transition probabilities per unit time $T_{\mathbf{i}'\mathbf{i}}$ in the following way:

We start by enumerating each of all possible configurations $\boldsymbol{i}=\{i_R,i_P\}$ of the population by an integer number — we do so by defining a function V such that $p=V(\mathbf{i})$ and $q=V(\mathbf{i}')$ and, therefore, $\boldsymbol{i}=V^{-\mathbf{1}}(p)$ and $\boldsymbol{i}'=V^{-\mathbf{1}}(q)$. Then, we may write $W_{qp}=T_{\mathbf{i}'\mathbf{i}}$. The transition probabilities $T_{\mathbf{i}'\mathbf{i}}$ can all be written in terms of the following expression, which gives the probability that an individual with strategy $X\in\{\boldsymbol{C},\boldsymbol{D}\}$ in the sub-population $k\in\{R,P\}$ changes to a different strategy $Y\in\{\boldsymbol{C},\boldsymbol{D}\}$, both from the same sub-population k and from the other population l (that is $l=P$ if $k=R$, and $l=R$ if $k=P$):

$$T_k^{X\to Y}=\frac{i_k^X}{Z}\left((1-\mu)\left[\frac{i_k^Y}{Z_k-1+(1-h)Z_l}\left(1+e^{\beta\left(f_k^X-f_k^Y\right)}\right)^{-1}+\frac{(1-h)i_l^Y}{Z_k-1+(1-h)Z_l}\left(1+e^{\beta\left(f_k^X-f_l^Y\right)}\right)^{-1}\right]+\mu\right).$$

Thus, when homophily is maximum ($h=1$) the imitation occurs only between individuals of the same wealth class (*rich* or *poor*), while when $h=0$ everyone influences and may be influenced by anyone else.

Another central quantity — which portrays the overall (stochastic) evolutionary dynamics in the space of all possible configurations — is the *gradient of selection* (**GoS**). For each configuration $\mathbf{i}=\{i_R,i_P\}$, we compute the most likely path each subpopulation $k\in\{R,P\}$ will follow, resorting to the probability to increase (decrease) by one, in each time-step, the number of Cooperators for that configuration $\mathbf{i}$ of the population; denoting this quantity by $T_{\mathbf{i},k}^{+}$ ($T_{\mathbf{i},k}^{-}$), we may write

$$\nabla_{\mathbf{i}}=\left\{T_{\mathbf{i},R}^{+}-T_{\mathbf{i},R}^{-},T_{\mathbf{i},P}^{+}-T_{\mathbf{i},P}^{-}\right\}$$

Finally, for each possible configuration $\mathbf{i}$, we make use of multivariate hypergeometric sampling (Equation **2** of main text) to compute the (average) fraction of groups that reach a total of $M\langle c\rangle$ in contributions, that is, that successfully achieve the public good — which we designate by $\eta(\mathbf{i}) \equiv \eta(i_R, i_P)$. The population average group achievement — η_G — is then computed by averaging over all possible configurations **i,** each weighted with the corresponding stationary distribution: $\eta_G = \sum_{\mathbf{i}} \bar{p}_{\mathbf{i}}\, \eta(\mathbf{i})$.

2. Population size dependence

Figure **S1** shows the behavior of η_G as a function of the population size Z, maintaining the ratio 4:1 between *poor* and *rich* and the same parameters of Figure **2** in main text, except for Z and $\mu = Z^{-1}$.

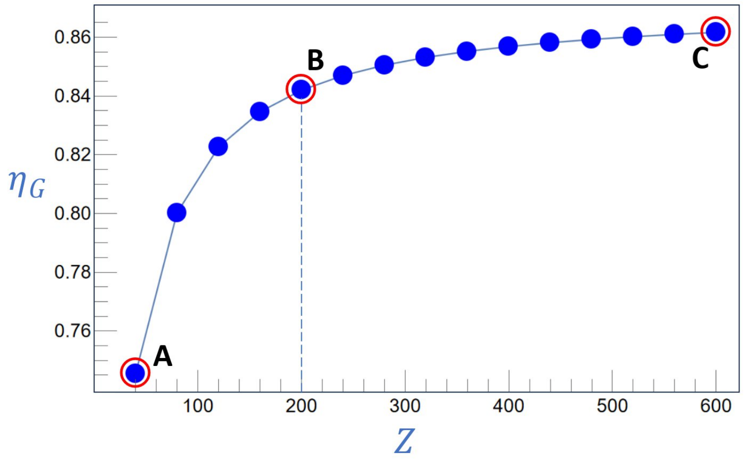

Figure S1. The population average group success η_G (see Methods for definition) is plotted as a function of the population size. We maintain the ratio 4:1 between *poor* and *rich* and keep all model parameters values used in Figure **2** of main text, except Z and $\mu = Z^{-1}$. The circles labeled **A**, **B** and **C** indicate the phase portraits in panels **A**, **B** and **C**, respectively, of Figure **S2**.

As one can observe, for $Z = 200$ (indicated with a vertical dashed line, the value used throughout this work), η_G is already in the region of slow convergence towards the large population size limit.

In Figure **S2** we show, in panels **A**, **B** and **C**, phase portraits of the co-evolutionary dynamics for the population sizes labeled with the same letters in Figure **S1**. Direct comparison of the phase portraits for different population sizes shows that the overall co-evolutionary dynamics remains qualitatively the same, with finite size effects becoming less important with increasing population size. As a result, $Z = 200$ proves to be a convenient choice both in terms of the number of countries worldwide, but also as a size at which small population size effects no longer affect the phase portraits (and corresponding quantities, such as η_G) in a significant way.

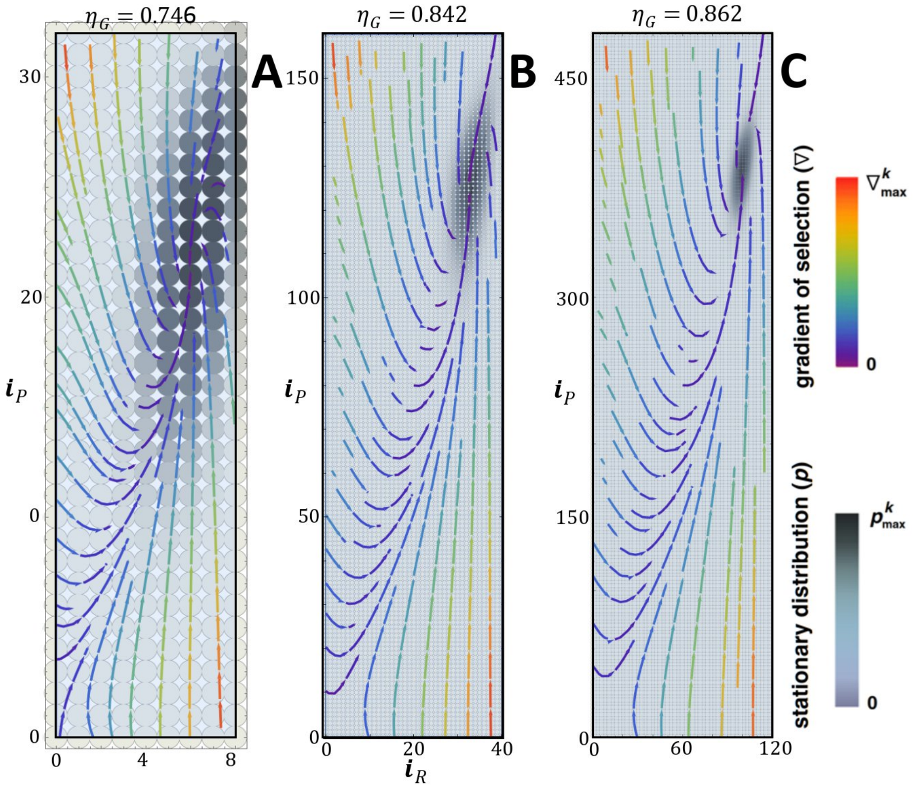

Figure S2. In all panels we employ the parameters used in Figure **S1**. **A.** Phase portraits of the co-evolutionary dynamics for $Z = 40$. **B.** Same as **A** for $Z = 200$ (cf. Figure **2** of main text). **C.** Same as **B** for $Z = 600$. Plot limits: $p^k_{max} = 22.9 \times 10^{-3}$ (**A**), $p^k_{max} = 5.3 \times 10^{-3}$ (**B**) and $p^k_{max} = 1.8 \times 10^{-3}$ (**C**); $\nabla^k_{max} = 17.85 \times 10^{-2}$ (**A**), $\nabla^k_{max} = 16.14 \times 10^{-2}$ (**B**) and $\nabla^k_{max} = 15.86 \times 10^{-2}$ (**C**).

3. A different partition of the population between Developed and Developing countries

The rationale for the Developed/Developing partition of the population employed in the main text relied on the fact that, by separating the population into 2 different wealth classes, a ratio of 4:1 between developing (*poor*) and developed (*rich*) countries followed the rule that 20% of wealthiest countries generate the same **GDP** as the remaining 80% of the countries worldwide. This, in our opinion, constitutes a “natural choice”, in line with was was done in (5).

Needless to say, other partitions are possible, and in order to check the sensitivity of the model to variations of its parameters, here we shall consider an alternative partition of the population into rich and poor classes, where we make $Z_R = Z_P = Z/2$. Such a scenario maybe of relevance if we take into consideration that several behavioral experiments were carried out employing equal number of rich (high endowment) and poor (low endowment) participants – for a recent exception see e.g., (8). Figure **S3** shows an analysis similar to the one carried out in Figure **2** of main text, but now for $Z_R = Z_P = 100$. Similar to the “natural choice”, we observe that the co-evolutionary dynamics remains dominated by an interior attractor, located in a region of low risk (and high success), where the population spends most of the time. Furthermore, we re-obtain the result that the co-evolutionary dynamics is sensitive to the rate of

decline of risk with increasing cooperation (σ) mostly for the developed countries, being insensitive to that rate for developing countries.

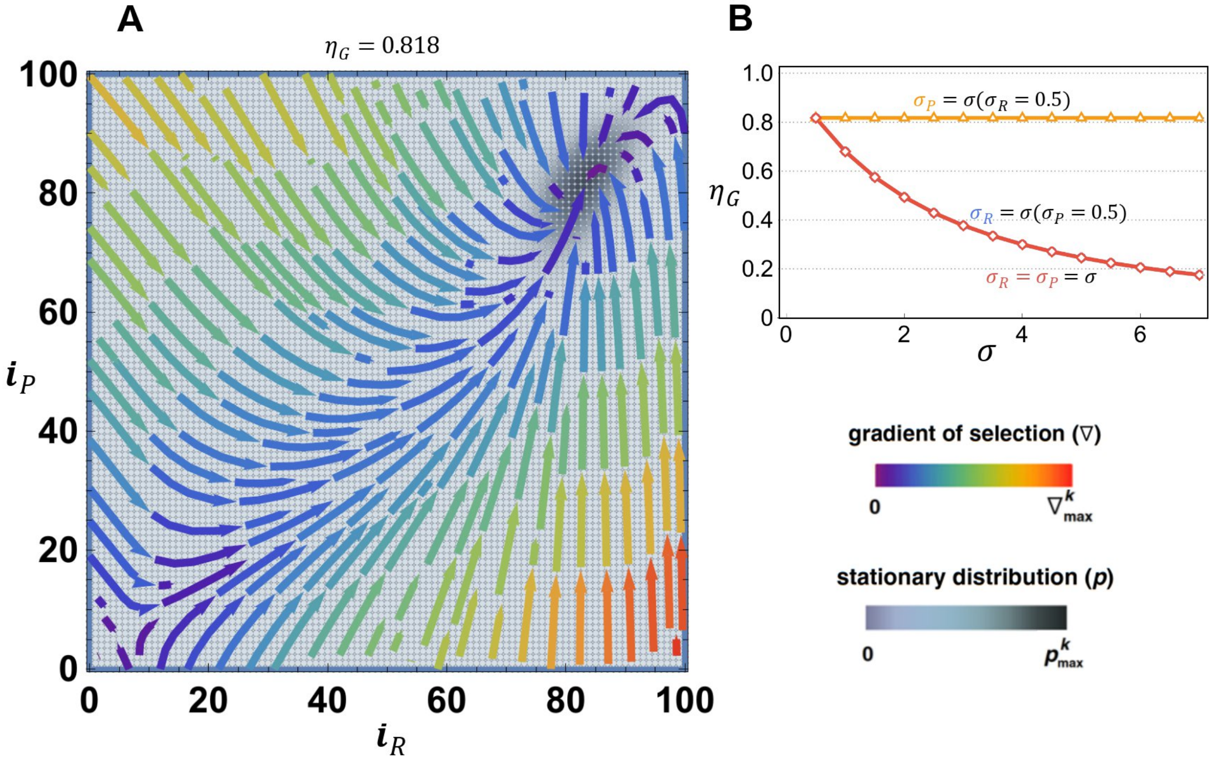

Figure S3. **Co-evolution of Cooperation and Risk in the CRD**. We employ the same notation of Figure **2** of main text. **A**. Stationary distribution and gradient of selection in the absence of homophily($h = 0$). The panel contains all possible configurations of the population (a total of $(1 + Z_R)(1 + Z_P)$), each specified by the number of *rich* (i_R) and *poor* (i_P), where in this case we have $Z_R = Z_P = 100$. The arrows show the gradient of selection (∇). The co-evolutionary dynamics is dominated by an attractor – centered at a configuration of the population in which ~83% of *rich* and ~82% of *poor* contribute to mitigate – and into which the population configuration converges in a stochastic sense, determining a final population average group success η_G ~82%. **B.** η_G is plotted as a function of the parameter σ controlling the rate of change of *risk* as a function of group success η (for fixed $\eta_{cut} = 0.0$). Here we allow σ to assume different values for *rich* (σ_R) and *poor* (σ_P) and compute η_G for situations in which we vary only σ_R (blue), only σ_P (orange) and both σ_R and σ_P (red). Clearly, the overall co-evolutionary dynamics is controlled by the value of σ_R, being insensitive to variations of σ_P . Model parameters:
$Z = 200, Z_R = 100, Z_P = 160,\ c_R = 0.17, c_P = 0.02, b_R = 1.7, b_P = 0.3, N = 6,\ M = 3, \beta = 5, \mu = Z^{-1}, h = 0,\ \eta_{cut} = 0,$
$\sigma_R = \sigma_P = 0.5$. Plot limits: $p^k_{max} = 6.07 \times 10^{-3}$; $\nabla^k_{max} = 25.21 \times 10^{-2}$.

The parameters of Figure **S3** were chosen to make it easily comparable with Figure **S2** in (5) where a value $\eta_G = 0.6$ was obtained for fixed $r = 0.3$. In other words, a partition $Z_R = Z_P$ requires a value of fixed risk higher than 0.3 in order to obtain η_G values comparable to what one obtains under variable risk. Compared to the "natural" partition employed in the main text, Figure **S3B** shows, intuitively, that a smaller fraction of Developing countries increases the insensitivity of the co-evolutionary dynamics to variations in σ_P.

4. Threshold considerations and co-evolutionary dynamics

In the main text, we parametrized the **CRD** in such a way that a direct comparison with (5) – Ref.(10) in main text – was possible. This way, and using (5) as a base scenario, we could directly assess the impact of the co-evolutionary dynamics under variable risk with that associated with fixed risk. Among other features, we found that under adaptive risk as implemented here one does not lose the feasibility of the emergence of overall cooperation in the **CRD**.

In the following, we shall consider in detail the implications of the cost of mitigation and of the threshold interpretation on the evolutionary dynamics in the **CRD** under adaptive risk.

As stated already, the underlying rationale for the 1/4 Developed/Developing partition used in the main text followed the rule that 20% of wealthiest countries generate the same **GDP** as the remaining 80% of the countries worldwide. By "defining proportional costs" via the cost factor c (5), we kept the cost to endowment ratio the same for both *rich* and *poor* countries, and defined the critical size for each group to successfully generate a public good by the condition (see Methods)

$$c_R\, j_R + c_P\, j_P \geq M\langle c\rangle \qquad \text{(S3)}$$

where j_R is the number of *rich* cooperators and j_P the number of *poor* cooperators in a group of size N; c_R is the cost of mitigation contributed by the *rich*, c_P the cost of mitigation contributed by the *poor*, whereas $\langle c\rangle$ is the population average cost of mitigation. Finally, we defined $0 \leq M \leq N$ as an integer number (see next section), in analogy with the standard definitions of a threshold public goods game (6, 7).

A recent assessment (see (8) and references therein) found that *rich* countries pay a cost to abate a given amount of CO_2 which is typically a factor $s \approx 3$ higher than the cost paid by *poor* countries to abate the same amount. If we take this (more realistic) estimate into account, Equation S3 may still apply, only that now we have that $c_R = 3c_P$ which, in turn, implies a small decrease of the average population cost $\langle c\rangle$ (keeping $c_P = 0.1b_P$ fixed, we get a reduction of 12.5% for $\langle c\rangle$). In such a framework, one expects that global cooperation will increase, and this is confirmed in Figure **S4**, where we compare the phase portraits of the co-evolutionary dynamics obtained for "proportional costs" (Main text, $s = 4$, Figure **S3A**) and those obtained employing the more realistic estimate using $c_R = 3c_P$ in Equation S3 ($s = 3$, Figure **S4C**).

Needless to say, the threshold condition may be defined in alternative ways. Instead of Equation S3 we may require, in line with the more standard threshold public goods game definition (6, 7), that the number of cooperators in a group of size N is at least M, replacing Equation S3 by Equation S4 below

$$j_R + j_P \geq M \qquad \text{(S4)}$$

For s =3, the threshold condition in Equation S4 means that M defines the minimum abatement in a group required to produce a Public Good. Naturally, this condition for $s = 4$ may be viewed as a scenario in which *rich* will pay more than *poor* for the same amount of abatement. Intuitively, one expects that the results of using Equation S4 will lead to less overall cooperation compared to those using Equation S3 (for the same value of s), and this may be confirmed by comparing the different panels in Figure **S4**, where results are shown for both $s = 4$ (panels **A** and **B**) and $s = 3$ (panels **C** and **D**) employing both Equation S3 (panels **A** and **C**) and Equation S4 (panels **B** and **D**).

Overall, and besides what was already discussed regarding the effect of parameter s, we notice that Equation S3 leads to a smaller dispersion of the stationary distribution for the *rich* in the vicinity of its maximum, whereas Equation S4 leads to an overall reduction of the average number of *poor* countries that cooperate.

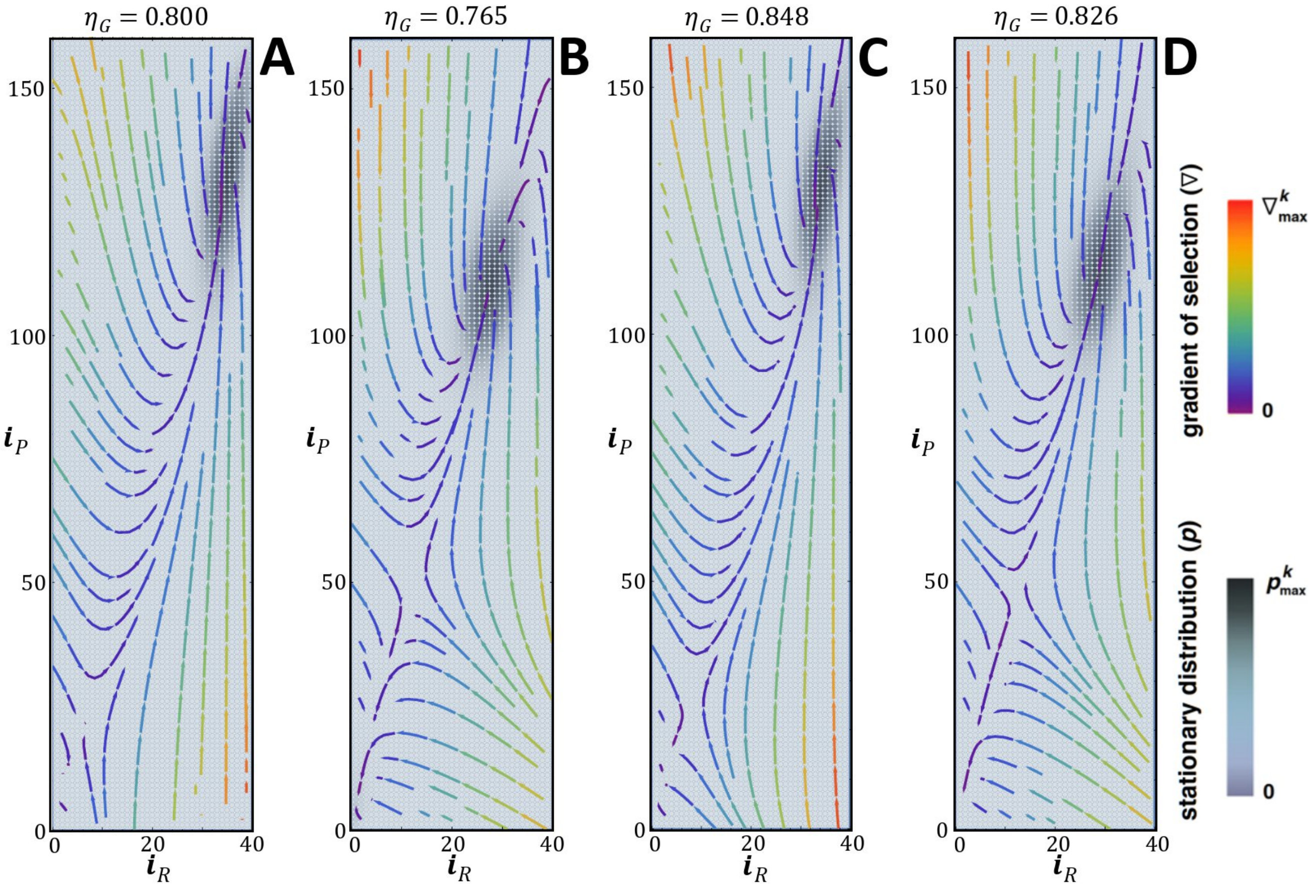

Figure S4. Phase portraits of the co-evolutionary dynamics for $Z = 200, Z_R = 40, Z_P = 160$, , $N = 8$, $M = 5, b_R = 4, b_P = 2.5, c_P = 0.1 b_P$, $c_R = s\, c_P$, $\langle b \rangle = 1, \beta = 3, \mu = Z^{-1}, h = 0$, $\eta_{cut} = 0, \sigma = 0.5$.
All these parameters remain the same for all panels. Panel specific parameters: **A** : $s = 4$, threshold Equation S3. **B** : $s = 4$, threshold Equation S4. **C** : $s = 3$, threshold Equation S3. **D** : $s = 3$, threshold Equation S4. In all cases we observe the same topological structure of the co-evolutionary dynamics, while threshold Equation S3 leads to peak stationary distributions of *rich* that are narrower than those originating from threshold Equation S4. Plot limits: $p^k_{max} = 55.7 \times 10^{-4}$ (**A**), $p^k_{max} = 48.9 \times 10^{-4}$ (**B**),, $p^k_{max} = 57.65 \times 10^{-4}$ (**C**) and $p^k_{max} = 49.4 \times 10^{-4}$ (**D**); $\nabla^k_{max} = 16.26 \times 10^{-2}$ (**A**), $\nabla^k_{max} = 16.37 \times 10^{-2}$ (**B**), $\nabla^k_{max} = 15.41 \times 10^{-2}$ (**C**) and $\nabla^k_{max} = 16.37 \times 10^{-2}$ (**D**).

Perhaps most importantly, the topological structure of the co-evolutionary dynamics remains unaffected by these different implementations of the model.

In what follows we shall employ $s = 3$ together with Equation S4 to discuss the dependence of the co-evolutionary dynamics on the threshold integer M. Needless to say, the results for other parametrizations will remain qualitatively similar.

5. Threshold dependence and the coupling between risk and cooperation

Changing the threshold value M imposes sizable changes in the overall game co-evolutionary dynamics. Because changing M also defines the minimum number of individuals in a group required to produce some public good (such that, and only in this case, a benefit will be made available to others), the behavior of η also changes significantly for different values of M, as shown in Figure **S5**.

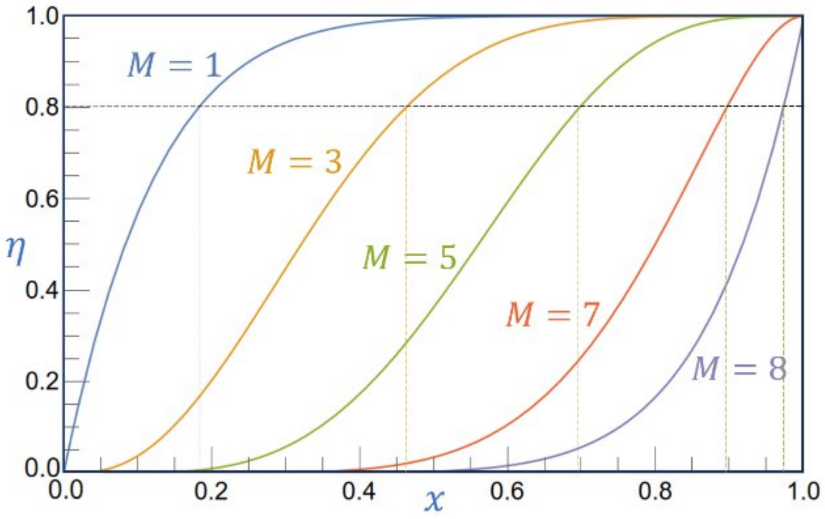

Figure S5. We plot the dependence of $\eta(x) -$ where x represents the fraction of cooperators in a population$-$ as a function of the group threshold M for groups of size $N = 8$. Because M dictates the number of individuals in the group required to produce a public good (we use Equation S4), the smaller the value of M the smaller the critical value x^* of the fraction cooperators in the population at which $\eta(x^*)$ reaches a specific value. This different behavior of $\eta(x)$ – and η_G – as a function of M must be taken into consideration in the analysis of the phase portraits depicted in the Figure **S6** below.

Figure **S5** shows the dependence of η on M for the case of a single wealth class, showing that high values of η are reached at very different fractions of cooperators in the population, for different M. Thus, and because the coupling defined here is between r and η, the risk "follows" η instead of assuming some pre-defined behavior as a function of the number (or fraction) of cooperators in the population.

As a result, the model developed here is conceptually different from the "adaptive feedback" approach developed in (9) (Ref.(19) of main text), where a pre-defined dependence on the fraction of cooperators in the population was assumed, in order to establish the coupling between risk and cooperation. To the best of our knowledge, Ref. (9) is the only one that, similar to our work, couples risk with cooperation.

A comparison between the present coupling between risk and cooperation and the framework investigated in (9) is non-trivial although, qualitatively, the present model resembles more the "exponential feedback" coupling model in (9). Furthermore, it is worth pointing out that our model, by dividing the population into 2 wealth classes, by allowing for homophilic behavior, and by performing a stochastic evolutionary dynamics in finite populations, greatly departs from that of Ref. (9).

Taking into account the results shown in Figure **S5**, in Figure **S6A** we show a typical phase portrait of the co-evolutionary dynamics for larger groups with $N = 8$ and $M = 5$, $s = 3$ and threshold Equation S4.

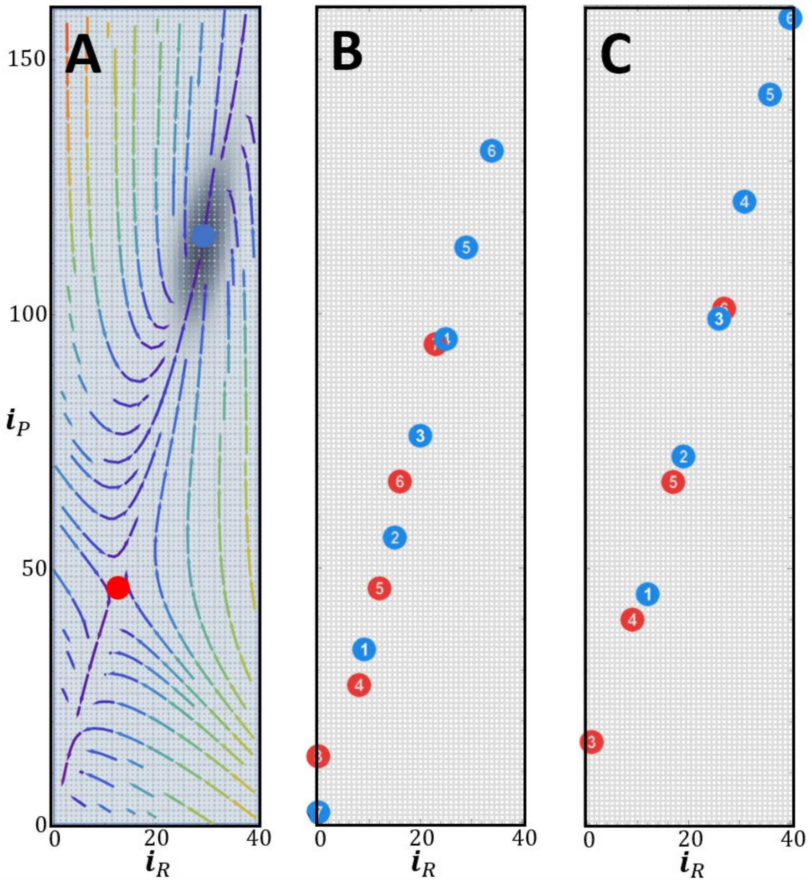

Figure S6. **A.** Phase portraits of the co-evolutionary dynamics for $Z = 200, N = 8, M = 5,\ c_P = 0.1\ b_P, c_R = 3\ c_P$ and $b_R = 4 \times b_P = 2.5$, employing threshold Equation S4. All other parameters as in Figure **2** of main text. We represent the location of the attractor by a solid blue circle, whereas the location off the saddle-like structure is indicated by the solid red circle. The phase portrait clearly exhibits the saddle/attractor dual structure that pervades throughout most of the parameter regions investigated. **B.** For the same parameters of **A** we now plot the location of the attractor (blue circles) and the saddle (red circles) for different values of the group threshold M. One can identify the value of M by the number inserted in each circle. Whenever no numbered circle appears, this means that the attractor/saddle is not present in the simplex. Increasing M helps to shift the location of both the attractor and the saddle "up right" towards increasing levels of cooperation, (qualitatively) along the main diagonal of the simplex, but up to a point: For $M = 7$ (in fact, for $M \geq 7$), the saddle-like structure shifts so much "up right" that the attractor appears now close to the configuration of full defection, with a corresponding collapse of cooperation. In keeping with this parametrization, cooperation also collapses for $N = M = 7$ (not shown). **C**. Same as **B** for $N = 6$, showing that, in this case, cooperation does not collapse for $M = N$ – instead, the population is driven now into full cooperation.

All other parameters remain unchanged with respect to Figure **2** of main text. As the group becomes larger ($N = 8$, compared to N =6 in Figure **2**), besides the attractor already discussed at length in the main text – here depicted with a solid blue circle – one can clearly observe the existence of a saddle-like structure at low values of cooperation (for both *rich* and *poor*, already pointed out in the main text),

which acts mostly as a probability repeller into states characterized either by no cooperation or by high-levels of cooperation (mostly for *rich*, and also partly for *poor*). This point becomes here clearly visible and is represented by a solid red circle.

The dual occurrence of these attractor and saddle-like structures pervades throughout most of the parameter regions of the model. However, while in most cases the attractor is clearly visible in the simplex, the same does necessarily happen with the saddle-like structure. For instance, for the parameters used in the main text (e.g., Figure **2**) the saddle-like structure is only present whenever $M \geq 3$ (Figure **S6C**).

The locations of the attractor (blue circles) and saddle-like structures (red circles) are shown in Figure **S6B** for different values of M, keeping $N = 8$. The value of M with which a given circle (red or blue) is associated is inserted in the circle. Figure **S6B** shows the general pattern observed: Increasing M helps to shift the location of both the attractor and the saddle "up right" into regions of high cooperation, (qualitatively) along the main diagonal of the simplex, but up to a point: For $M = 7$ (in fact, for $M \geq 7$), the saddle-like structure shifts so much "up" that the attractor appears now close to the configuration of full defection, at which point cooperation collapses. In keeping with this parametrization, cooperation also collapses for $N = M = 7$, although it remains high for $N = 7$ and $M = 6$ (not shown). Figure **S6C** shows the dependence on M for groups of size $N = 6$. In this case, the limit $N = M = 6$ essentially leads to full cooperation in the population. In fact, it turns out that $N = 6$ is the largest group size for which cooperation does not collapse when $M = N$. With increasing group size N the highest value of M for which cooperation survives decreases. Clearly, the results discussed above apply for the case of $\sigma = 0.5$. With increasing σ the impact on overall cooperation is sizable, as one can easily infer from Figure **2B** of main text which, in turn, lowers the critical M-values for the survival of cooperation.

SI References

1. G. Szabó, C. Toke, Evolutionary prisoner's dilemma game on a square lattice. *Phys Rev E* **58**, 69-73 (1998).
2. A. Traulsen, M. A. Nowak, J. M. Pacheco, Stochastic dynamics of invasion and fixation. *Phys. Rev. E* **74**, 011909 (2006).
3. N. V. Kampen, *Stochastic processes in physics and chemistry*, North-Holland Personal Library (North-Holland, Amsterdam, The Netherlands, 2007).
4. L. A. Imhof, D. Fudenberg, M. A. Nowak, Evolutionary cycles of cooperation and defection. *Proc Natl Acad Sci U S A* **102**, 10797-10800 (2005).
5. V. V. Vasconcelos, F. C. Santos, J. M. Pacheco, S. A. Levin, Climate policies under wealth inequality. *Proc Natl Acad Sci U S A* **111**, 2212-2216 (2014).
6. J. M. Pacheco, F. C. Santos, M. O. Souza, B. Skyrms, Evolutionary dynamics of collective action in N-person stag hunt dilemmas. *Proc Biol Sci* **276**, 315-321 (2009).
7. M. O. Souza, J. M. Pacheco, F. C. Santos, Evolution of cooperation under N-person snowdrift games. *Journal of theoretical biology* **260**, 581-588 (2009).
8. Y. Dong, S. Ma, B. Zhang, W.-X. Wang, J. M. Pacheco, Financial incentives to poor countries promote net emissions reductions in multilateral climate agreements. *One Earth* **4**, 1141-1149 (2021).
9. L. Liu, X. Chen, A. Szolnoki, Coevolutionary dynamics via adaptive feedback in collective-risk social dilemma game. *eLife* **12**, e82954 (2023).
